# Advanced age is associated with increased adverse outcomes in patients undergoing middle cerebral artery stenting

**DOI:** 10.3389/fneur.2022.1037034

**Published:** 2023-01-18

**Authors:** Gong Wang, Juan Liu, Shengrong He, Xi Zhang, Libai Yang, Fei Gao, Yu Guo, Rui Xu

**Affiliations:** ^1^Department of Neurology, The Second Affiliated Hospital, Army Medical University, Chongqing, China; ^2^Department of Field Internal Medicine, The Second Affiliated Hospital, Army Medical University, Chongqing, China; ^3^Department of Radiology, The Second Affiliated Hospital, Army Medical University, Chongqing, China; ^4^Department of Neurology, The Second People's Hospital of Banan District, Chongqing, China

**Keywords:** age, MCA stenting, complications, risk factors, retrospective study

## Abstract

**Purpose:**

This study tried to evaluate whether advanced age has an increased incidence of major complications in patients undergoing MCA stenting.

**Methods:**

A total of 348 patients who underwent MCA stenting were reviewed from a prospectively maintained database. Ninety-day ischemic stroke, intracerebral hemorrhage, and death outcomes were compared among the young (≤40 years old), middle (41–60 years old) and old (≥61 years old) groups. Univariate analysis and multivariable logistic regression analysis were used to investigate different variables associated with 90-day major adverse events. Kaplan–Meier analysis was performed to determine long-term outcomes during follow-up.

**Results:**

The incidence of 90-day ischemic stroke was 9.26% in the old group, 2.86% in the middle group, and 0% in the young group (*P* = 0.024). The incidence of all 90-day major adverse events was 3.33% in patients ≤40 years old, 19.90% in patients 41–60 years old, and 24.07% in patients ≥61 years old, with statistical significance (*P* = 0.04). Advanced age was associated with increased 90-day ischemic stroke (OR = 1.074, 95% CI: 1.019–1.132, *P* = 0.007; adjusted OR: 1.071, 95% CI: 1.008–1.138, *P* = 0.026) and 90-day death (OR = 1.072, 95% CI: 1.012–1.135, *P* = 0.018; adjusted OR: 1.095, 95% CI: 1.015–1.182, *P* = 0.018). Meanwhile, advanced age was also associated with decreased long-term survival and ischemic stroke-free survival during follow-up.

**Conclusion:**

Our data indicated that MCA stenting in elderly patients is associated with a high risk of adverse events and should be cautiously considered.

## 1. Introduction

Intracranial large artery stenosis (ICAS) or occlusion is common, especially in patients of Asian, Black, and Hispanic ancestry (1), and the incidence of stroke recurrence is much greater with ICAS than without ICAS (15–22.1 vs. 5.3–5.5%, *P* < 0.01) (2–4). Thus, aggressive therapeutic strategies, such as dual antiplatelet treatment or percutaneous transluminal angioplasty and stenting (PTAS), have been adopted to reduce stroke recurrence in these patients. In the CHANCE study, researchers reported that, in patients with ICAS, the recurrence rate of stroke had almost the same high value in the mono-antiplatelet group as in the dual-antiplatelet group (13.6 vs. 11.3%, *P* > 0.05) (5). Failure of aggressive medical therapy for secondary prevention of stroke makes the PTAS approach a tantalizing prospect to reduce the recurrence of stroke in patients with ICAS.

Unfortunately, in the SAMMPRIS study, researchers reported that the use of aggressive medical management was superior to PTAS with the Wingspan system in high-risk patients with atherosclerotic intracranial arterial stenosis. One of the reasons was that PTAS increased the occurrence of adverse events, such as peri-operative ischemic or hemorrhagic stroke (6). However, researchers in the SAMMPRIS study also emphasized that further randomized trials assessing new techniques that might substantially lower the risk of the most frequent types of periprocedural strokes are still needed. Therefore, neural interventional groups around the world are still trying to find a way to lower the procedural risk, for instance, using new stents (4) or identifying risk factors for periprocedural strokes, which further helps to identify any subgroup that may benefit from PTAS.

Specifically, the conclusion of endovascular treatment for middle cerebral artery stenosis (MCAS) is controversial. Miao et al. (7) reported that endovascular treatment is as safe but not better than medical treatment for symptomatic MCAS. However, a single-center study reported that PTAS of the middle cerebral artery (MCA) was safe and associated with a low periprocedural complication rate (8). Based on the controversial results, it is important to identify risk factors when PTAS is used for the treatment of MCAS, as well as to guide patient selection in clinical practice.

Aging is the process of functional decline in cells, tissues and organs (9). In carotid artery stenting, it was reported that age status affects periprocedural outcome, and evidence suggests an increased risk with increasing patient age (10–13). To our knowledge, there is a lack of research assessing whether age affects the periprocedural and long-term effects of PTAS for patients with MCAS.

Our goal in this study was to evaluate our retrospectively maintained database of patients who had undergone MCA stenting to determine whether age affects periprocedural and long-term outcomes during the follow-up period.

## 2. Material and methods

### 2.1. Patients

We retrospectively analyzed 348 patients with MCAS who had been treated with MCA stenting from March 2005 to July 2020 using the stroke database of the Department of Neurology at the Second Affiliated Hospital of Army Medical University. In this study, most of patients included were from ASSIST clinical trial (trial registration: Chinese Clinical Trial Registry: ChiCTR1800015766) and EPZ clinical trial (trial registration: Chinese Clinical Trial Registry: ChiCTR1900027538). A few patients were not involved in any clinical trial, but these patients were all symptomatic intracranial atherosclerotic stenosis which could not be well controlled with double antiplatelet therapy. In addition, benefits and risks of the operation were informed to the patient in detail before the operation and written informed consent for operation was obtained.

The inclusion criteria were as follows: (1) recurrence of transient ischemic attack (TIA), mild or moderate ischemic stroke (National Institutes of Health Stroke Scale, NIHSS ≤ 12) within the previous 180 days, despite antiplatelet therapy. And stenting was placed after clinical stabilization (at least for 1 week after acute ischemic stroke); (2) digital subtraction angiography-verified severe atherosclerotic stenosis (≥70%) (14) or occlusion in the M1 segments of the MCA, using the method of the North American Symptomatic Carotid Endarterectomy Trial (NASCET) or the WASID measurement (if the distal vessel was not accessible) (4); (3) the previous stroke occurring more than 7 days before stenting; (4) age between 20 and 80 years old; (5) a drop in cerebral blood flow by 30% or more compared to the contralateral MCA circulation territory on computed tomography perfusion (CTP) imaging; and (6) at least one atherosclerotic risk factor (hypertension, diabetes mellitus, hyperlipidemia, hyperhomocysteinemia, and/or smoking).

The exclusion criteria were as follows: (1) a nonatherosclerotic lesion confirmed by high-resolution magnetic resonance imaging (HR-MRI); (2) two or more stents placed after percutaneous transluminal angioplasty (for patient with more than one stents, the length and site of lesion might be different, meanwhile the hemodynamics, the duration of operation and the stenting procedure were totally different); (3) concurrent intracranial pathology, including tumors, aneurysms, or arteriovenous malformation; (4) contraindication to heparin, aspirin, clopidogrel, or anesthetic; or (5) life expectancy of <1 year due to other medical conditions.

The study was approved by the Medical Ethics Committee of the Second Affiliated Hospital, Army Medical University. Written informed consent, including informed consent for operation and agreement of data analysis and publication, was obtained from every participant or from authorized family member before the operation. The study protocol was performed in accordance with relevant ethical guidelines and regulations for human studies.

### 2.2. Peri-operative management and stenting procedure

For patients involved in this retrospective study, coronary and cranial computed tomographic angiography, brain CTP imaging, and HR-MRI had been obtained before stenting. Routine oral aspirin (100 mg/day) and clopidogrel (75 mg/day) had been administered 3–5 days before stenting. The risk factors of hypertension, low density lipoprotein, homocysteine, and high blood glucose had been controlled according to published guidelines for the prevention of secondary ischemic stroke (15). Smoking and drinking had been also prohibited.

Three experienced neurointerventionists performed the stenting procedure, as previously reported (4). Briefly, after general anesthesia and systemic heparinization, femoral artery puncture was performed to place a 6F/8F arterial sheath. A 6F guiding catheter Envoy (Cerenovus, Fremont, CA, USA) was delivered to the C2–3 segment of the internal carotid artery. Over a Synchro-10 guidewire (Stryker Neurovascular, Fremont, CA, USA), an Echelon 10 microcatheter (Medtronic Neurovascular, CA, USA) was navigated to the distal part of the stenosis. The Synchro-10 guidewire was then retrieved, and an Exchangeable Synchro-14 guidewire (Stryker Neurovascular, Fremont, CA, USA) was advanced into the artery distal to the stenosis through the microcatheter. Then, a Gateway balloon (Boston Scientific, Natick, MA, USA) was placed across the stenotic segment for balloon dilatation. The choice of stent type (Wingspan or Neuroform EZ) was decided based on the diameter of the target artery and the site of the lesion (4). If no abnormalities were observed, the stenting procedure was completed.

After the operation, brain CT scanning was performed immediately after surgery to exclude brain hemorrhage. Blood pressure was controlled at 110–130/70–80 mmHg. If patients were free of hemorrhage, enoxaparin sodium was administered by subcutaneous injection at 40 mg/12 h for 3–5 days. Oral treatment with aspirin (100 mg/day) and clopidogrel (75 mg/day) was continued for at least 3 months, followed by long-term oral administration of either aspirin (100 mg/day) or clopidogrel (75 mg/day). Oral atorvastatin (40 mg/day) was also administered for at least 3 months.

Specifically, it should be clarified that we had to admit that there was an advancement in terms of the used materials and operator experience from 2005 to 2020, however, we had tried our best to ensure the consistency and homogeneity of our study: (1) We had a stable neurointervention team including three senior doctors, who performed and supervised the interventional surgery. (2) The choice of MCA stent type was limited to Wingspan or Neuroform EZ.

### 2.3. Data collection

The characteristics of the patients, comorbidities and current medications were recorded. All events during the 90-day recovery period and follow-up period were recorded (16, 17). Major adverse events included ischemic stroke (stent-related), intracerebral hemorrhage (ICH, confirmed by CT scan), transient ischemic attack (TIA), myocardial infarction (MI), and death (stroke-related, including death from complications caused by stroke). Specifically, ischemic stroke was confirmed by diffusion weighted imaging (DWI) or defined as an increase of ≥2 in the total NIHSS score or ≥1 in the motor NIHSS score (18) meanwhile ICH was ruled out by CT scan.

### 2.4. Statistical analysis

Statistical analysis was performed by using chi-square analysis for categorical variables. When chi-square analysis was not appropriate for less frequent occurrences, Fisher's exact test was performed. For continuous variables, such as time for qualifying event to stenting, one-way ANOVA was used to evaluate statistical significance. Univariate analysis was used to investigate demographic, clinical, and procedural variables for association with 90-day major adverse events (ICH, ischemic stroke and death). The possible confounding factors were further adjusted using a multivariable logistic regression analysis (19), all of the variables entered in the regression analysis based on their known association with outcome of interest and univariate analysis results, variables were excluded if *P* > 0.9 in univariate analysis. Kaplan–Meier curves for survival, long-term ischemic stroke-free survival, and long-term ICH survival were compared using the log rank test. Any value expressed as *P* < 0.05 was considered significant. Statistical analyses were conducted using GraphPad Prism 5.0 or SPSS 18.0.

## 3. Results

### 3.1. Baseline characteristics

Patient clinical characteristics are delineated in Table 1. The patients were divided into three groups according to previously reported methods (9) with minor modifications: ≤40 years of age (young), 41–60 years of age (middle) and ≥61 years of age (old). The old group was an average of 15.9 years older than the middle group, and the middle group was an average of 16.1 years older than the young group.

**Table 1:** Patient characteristics before middle cerebral artery stenting.

**Variables**	**No. (%) of patients**			***P*** **-value**
	≤ **40 years** **(34.6** ±**4.6**, ***n*** =**30)**	**41–60 years** **(50.7** ±**5.5**, ***n*** =**210)**	≥**61 years** **(66.6** ±**4.6**, ***n*** = **108)**	
**Sex**				0.013
Male	29 (96.67%)	159 (75.71%)	76 (70.37%)	
Female	1 (3.33%)	51 (24.29%)	32 (29.63%)	
Hypertension	7 (23.33%)	137 (65.24%)	83 (76.85%)	0.000
CAD	1 (3.33%)	17 (8.10%)	28 (25.93%)	0.000
MI	0 (0.00)	0 (0.00)	1 (0.93%)	0.397
Atrial fibrillation	0 (0.00)	2 (0.95%)	2 (1.85%)	0.726
COPD	0 (0.00)	0 (0.00)	1 (0.93%)	0.397
Diabetes mellitus	0 (0.00)	84 (40.00%)	45 (41.67%)	0.000
Homocysteine	11 (36.67%)	95 (45.24%)	70 (64.81%)	0.001
Hyperlipidemia	15 (50.00%)	107 (50.95%)	46 (42.59%)	0.363
Smoking history	16 (53.33%)	69 (32.86%)	40 (37.04%)	0.089
History of TIA	13 (43.33%)	51 (24.29%)	15 (13.89%)	0.002
History of ischemic stroke	14 (46.67%)	151 (71.90%)	89 (82.41%)	0.000
History of ICH	0 (0.00)	2 (0.95%)	1 (0.93%)	0.867
Symptomatic presentation	28 (93.33%)	192 (91.43%)	105 (97.22%)	0.126
**Stenosis site**				0.959
Proximal MCA	17 (56.67%)	121 (57.62%)	60 (55.56%)	
Mid MCA	11 (36.67%)	79 (37.62%)	42 (38.89%)	
Distal MCA	2 (6.67%)	10 (4.76%)	6 (5.56%)	
Time for qualifying event to stenting (day, mean ± standard deviation)	24.73 ± 31.33	20.62 ± 24.56	21.02 ± 26.65	0.717
**Year of procedure**				0.061
2005	0 (0.00)	1 (0.48%)	0 (0.00)	
2007	0 (3.33%)	1 (0.48%)	0 (0.00)	
2008	0 (0.00)	2 (0.95%)	1 (0.93%)	
2009	0 (3.33%)	2 (0.95%)	2 (1.85%)	
2010	2 (6.67%)	3 (1.43%)	3 (2.78%)	
2011	4 (13.33%)	7 (3.33%)	4 (3.70%)	
2012	5 (16.67%)	10 (4.76%)	5 (4.63%)	
2013	2 (6.67%)	13 (6.19%)	8 (7.41%)	
2014	0 (0.00)	18 (8.57%)	8 (7.41%)	
2015	1 (3.33%)	12 (5.71%)	8 (7.41%)	
2016	3 (10.00%)	13 (6.19%)	15 (13.89%)	
2017	5 (16.67%)	23 (10.95%)	9 (8.33%)	
2018	4 (13.33%)	36 (17.14%)	17 (15.74%)	
2019	2 (6.67%)	39 (18.57%)	15 (13.89%)	
2020	0 (0.00)	30 (14.29%)	13 (12.04%)	

CAD, coronary artery disease; MI, myocardial infarction; COPD, chronic obstructive pulmonary disease; TIA, transient ischemic attack; ICH, intracerebral hemorrhage.

For comorbidities, an increased incidence of hypertension (young vs. middle vs. old, 23.33 vs. 65.24 vs. 76.85%, *P* < 0.001), coronary artery disease (CAD, young vs. middle vs. old, 3.33 vs. 8.10 vs. 25.93%, *P* < 0.001), diabetes mellitus (young vs. middle vs. old, 0 vs. 40.00 vs. 41.67%, *P* < 0.001) and homocysteine (young vs. middle vs. old, 36.67 vs. 45.24 vs. 64.81%, *P* = 0.001) was found in the middle and old groups. Meanwhile, significant differences were noted in the TIA (young vs. middle vs. old, 43.33 vs. 24.29 vs. 13.89%, *P* = 0.001) and ischemic stroke (young vs. middle vs. old, 46.67 vs. 71.90 vs. 82.41%, *P* < 0.001) history among the three groups. No significant differences were found in stenosis site, time for qualifying events to stent or year of procedure.

Medication histories before MCA stenting were not significantly different among the three groups, except for the use of calcium-channel blockers (Supplementary Table S1).

During the follow-up (within 3 months and after 3 months), similar percentages of patients took antiplatelet medications (Supplementary Table S1). However, the use of statin anticholesterol medications was significantly different among the three groups at follow-up.

### 3.2. Ninety-day neurologic events and mortality

As shown in Table 2, the incidence of ischemic stroke (stent-related) during the 90-day recovery stage was 9.26% in patients ≥61 years old, 2.86% in patients 41–60 years old, and 0% in patients ≤40 years old (*P* = 0.024). Ninety-day death had a similar trend but without statistical significance: 7.41% for patients ≥61 years old, 2.38% for patients 41–60 years old, and 0% for patients ≤40 years old (*P* = 0.064).

**Table 2:** Major adverse events ≤90 days of stent procedure.

	**No. (%) of patients**			***P*** **-value**
	≤ **40 years** **(*****n*** =**30)**	**41–60 years** **(*****n*** =**210)**	≥**61 years** **(*****n*** = **108)**	
Ischemic stroke (stent related)	0 (0.00)	6 (2.86%)	10 (9.26%)	0.024
ICH	1 (3.33%)	29 (13.81%)	8 (7.41%)	0.117
TIA	0 (0.00)	0 (0.00)	0 (0.00)	N/A
In-stent restenosis (>50%)	0 (0.00)	0 (0.00)	0 (0.00)	N/A
MI	0 (0.00)	0 (0.00)	0 (0.00)	N/A
Death	0 (0.00)	5 (2.38%)	8 (7.41%)	0.064
Total	1 (3.33%)	40 (19.90%)	26 (24.07%)	0.04

ICH, intracerebral hemorrhage; TIA, transient ischemic attack; MI, myocardial infarction.

By grouping all major adverse events during the 90-day recovery stage, the incidence was 3.33% in patients ≤40 years old, 19.90% in patients 41–60 years old, and 24.07% in patients ≥61 years old, with statistical significance (*P* = 0.04).

In the subgroup analysis, patients with MCA occlusion who received intracranial stent implantation were analyzed. In this present study, only 3, 25 and 5 MCA occlusion patients were included in patients ≤40 years old, 41–60 years old and ≥61 years old, respectively (Supplementary Table S2). And 90-day outcome analysis showed that only two ICH, one death were found in patients 41–60 years old. The subgroup data was not suitable for statistical analysis due to the small sample size.

In MCA severe atherosclerotic stenosis subgroup analysis, the incidence of ischemic stroke during the 90-day stage was 9.62% in patients ≥61 years old, 3.24% in patients 41–60 years old, and 0% in patients ≤40 years old (*P* = 0.043, Supplementary Table S3). While 90-day mortality was also significant different: 7.69% for patients ≥ 61 years old, 2.16% for patients 41–60 years old, and 0% for patients ≤40 years old (*P* = 0.049, Supplementary Table S3).

### 3.3. Univariable and multivariable analyses

Univariable analysis was used to investigate demographic, clinical, and procedural variables for association with 90-day ischemic stroke, ICH, and death, while confounding factors were adjusted using multivariable logistic regression. As shown in Table 3, patients with increased age were associated with increased ischemic stroke (univariable analysis: OR = 1.074, 95% CI: 1.019–1.132, *P* = 0.007; multivariable analysis: OR = 1.071, 95% CI: 1.008–1.138, *P* = 0.026). Hypertension was a risk factor for ischemic stroke when using univariable analysis (OR = 8.491, 95% CI: 1.108–65.075, *P* = 0.04), while the association was not observed after adjusting for confounding factors (OR = 6.194, 95% CI: 0.768–49.956, *P* = 0.087).

**Table 3:** Association among 90-day ischemic stroke and selected demographic, procedural, and clinical factors.

**Factors**	**Univariable analysis**		**Multivariable analysis^a^**	
	**OR (95%CI)**	***P*** **-value**	**OR (95%CI)**	***P*** **-value**
**Age**	**1.074 (1.019–1.132)**	**0.007**	**1.071 (1.008–1.138)**	**0.026**
**Hypertension**	**8.491 (1.108–65.075)**	**0.040**	6.194 (0.768–49.956)	0.087
CAD	2.342 (0.923–5.946)	0.073	1.916 (0.635–5.776)	0.248
Diabetes mellitus	1.069 (0.379–3.014)	0.900	Excluded	
Homocysteine	0.976 (0.358–2.662)	0.962	Excluded	
Hyperlipidemia	1.825 (0.648–5.136)	0.255	2.250 (0.725–6.979)	0.160
Smoking history	0.762 (0.259–2.246)	0.623	0.686 (0.151–3.112)	0.625
Drinking history	0.787 (0.286–2.163)	0.643	1.067 (0.251–4.530)	0.930
History of ischemic stroke	2.683 (0.598–12.037)	0.197	2.236 (0.296–16.899)	0.435
Symptomatic presentation	1.221 (0.155–9.631)	0.849	0.340 (0.022–5.310)	0.441
Aspirin use	1.387 (0.301–6.387)	0.674	3.566 (0.374–34.007)	0.269
Clopidogrel use	0.819 (0.104–6.455)	0.849	0.258 (0.015–4.597)	0.357
Time for qualifying event to stenting	1.008 (0.995–1.020)	0.224	1.008 (0.995–1.021)	0.233

CAD, coronary artery disease; OR, odds ratio; CI, confidence interval.

a Adjusted for hypertension (or age), CAD, hyperlipidemia, smoking history, drinking history, history of ischemic stroke, symptomatic presentation, aspirin use, clopidogrel use, time for qualifying event to stenting.

Age was analyzed as an independent, continuous variable. The bold values were considered significant with *P* < 0.05.

Only one variable had a statistically significant association with ICH: hypertension. Patients with hypertension were associated with increased ICH (univariable analysis: OR = 3.946, 95% CI: 1.499–10.393, *P* = 0.005; multivariable analysis: OR = 4.943, 95% CI: 1.788–13.663, *P* = 0.002; Table 4).

**Table 4:** Association among 90-day ICH and selected demographic, procedural, and clinical factors.

**Factors**	**Univariable analysis**		**Multivariable analysis^a^**	
	**OR (95%CI)**	***P*** **-value**	**OR (95%CI)**	***P*** **-value**
**Hypertension**	**3.946 (1.499–10.393)**	**0.005**	**4.943 (1.788–13.663)**	**0.002**
Age	0.985 (0.954–1.016)	0.332	0.966 (0.929–1.004)	0.079
CAD	0.926 (0.363–2.360)	0.871	1.069 (0.412–2.773)	0.892
Diabetes mellitus	0.907 (0.446–1.842)	0.786	1.025 (0.476–2.204)	0.950
Homocysteine	0.866 (0.441–1.700)	0.676	0.927 (0.444–1.934)	0.839
Hyperlipidemia	1.734 (0.872–3.450)	0.117	1.839 (0.888–3.811)	0.101
Smoking history	1.121 (0.562–2.236)	0.745	2.222 (0.880–5.611)	0.091
Drinking history	0.718 (0.363–1.419)	0.341	0.464 (0.187–1.151)	0.098
History of TIA	0.746 (0.315–1.767)	0.506	0.956 (0.288–3.169)	0.941
History of ischemic stroke	0.897 (0.426–1.890)	0.776	0.934 (0.281–3.105)	0.912
Symptomatic presentation	0.480 (0.170–1.356)	0.166	0.458 (0.106–1.975)	0.295
Aspirin use	1.140 (0.378–3.439)	0.816	2.865 (0.526–15.604)	0.224
Clopidogrel use	0.662 (0.150–2.919)	0.586	0.314 (0.037–2.669)	0.289
Time for qualifying event to stenting	0.994 (0.976–1.012)	0.513	0.995 (0.978–1.012)	0.567

CAD, coronary artery disease; TIA, transient ischemic attack; OR, odds ratio; CI, confidence interval.

a Adjusted for age, CAD, diabetes mellitus, homocysteine, hyperlipidemia, smoking history, drinking history, history of TIA, history of ischemic stroke, symptomatic presentation, aspirin use, clopidogrel use, time for qualifying event to stenting.

Age was analyzed as an independent, continuous variable. The bold values were considered significant with *P* < 0.05.

Age status was the only risk factor for 90-day death after MCA stenting (univariable analysis: OR = 1.072, 95% CI: 1.012–1.135, *P* = 0.018; multivariable analysis: OR = 1.095, 95% CI: 1.015–1.182, *P* = 0.018; Table 5).

**Table 5:** Association among 90-day death and selected demographic, procedural, and clinical factors.

**Factors**	**Univariable analysis**		**Multivariable analysis^a^**	
	**OR (95%CI)**	***P*** **-value**	**OR (95%CI)**	***P*** **-value**
**Age**	**1.072 (1.012–1.135)**	**0.018**	**1.095 (1.015–1.182)**	**0.018**
Hypertension	1.208 (0.364–4.005)	0.758	0.857 (0.222–3.314)	0.823
CAD	1.675 (0.530–5.291)	0.380	1.138 (0.261–4.952)	0.864
Diabetes mellitus	1.549 (0.509–4.714)	0.441	1.710 (0.502–5.825)	0.391
Homocysteine	1.146 (0.377–3.482)	0.810	0.814 (0.242–2.734)	0.739
Hyperlipidemia	1.740 (0.558–5.429)	0.340	2.417 (0.681–8.578)	0.172
Smoking history	2.037 (0.669–6.199)	0.210	1.453 (0.279–7.564)	0.657
Drinking history	1.668 (0.535–5.204)	0.378	1.586 (0.283–8.876)	0.599
History of TIA	0.275 (0.035–2.145)	0.218	0.573 (0.051–6.439)	0.652
History of ischemic stroke	1.243 (0.335–4.619)	0.745	0.880 (0.100–7.739)	0.909
Symptomatic presentation	0.424 (0.089–2.025)	0.282	0.202 (0.016–2.564)	0.218
Aspirin use	3.050 (0.796–11.690)	0.104	5.856 (0.612–56.039)	0.125
Clopidogrel use	2.356 (0.494–11.244)	0.282	0.483 (0.037–6.389)	0.581
Time for qualifying event to stenting	0.997 (0.970–1.024)	0.808	0.998 (0.972–1.025)	0.890

CAD, coronary artery disease; TIA, transient ischemic attack; OR, odds ratio; CI, confidence interval.

a Adjusted for hypertension, CAD, diabetes mellitus, homocysteine, hyperlipidemia, smoking history, drinking history, history of TIA, history of ischemic stroke, symptomatic presentation, aspirin use, clopidogrel use, time for qualifying event to stenting.

Age was analyzed as an independent, continuous variable. The bold values were considered significant with *P* < 0.05.

In MCA severe atherosclerotic stenosis subgroup analysis, age and hypertension were risk factors for 90-day ischemic stroke when using univariable analysis (Supplementary Table S4), while the association was not observed after adjusting for confounding factors (Supplementary Table S4). However, age was still the only risk factor for 90-day death after MCA stenting in this subgroup (univariable analysis: OR = 1.081, 95% CI: 1.016–1.150, *P* = 0.013; multivariable analysis: OR = 1.126, 95% CI: 1.030–1.232, *P* = 0.009; Supplementary Table S5). Meanwhile, hypertension still had a statistically significant association with ICH in the subgroup analysis (Supplementary Table S6).

### 3.4. Late survival and freedom from adverse events

**Figure 1:**
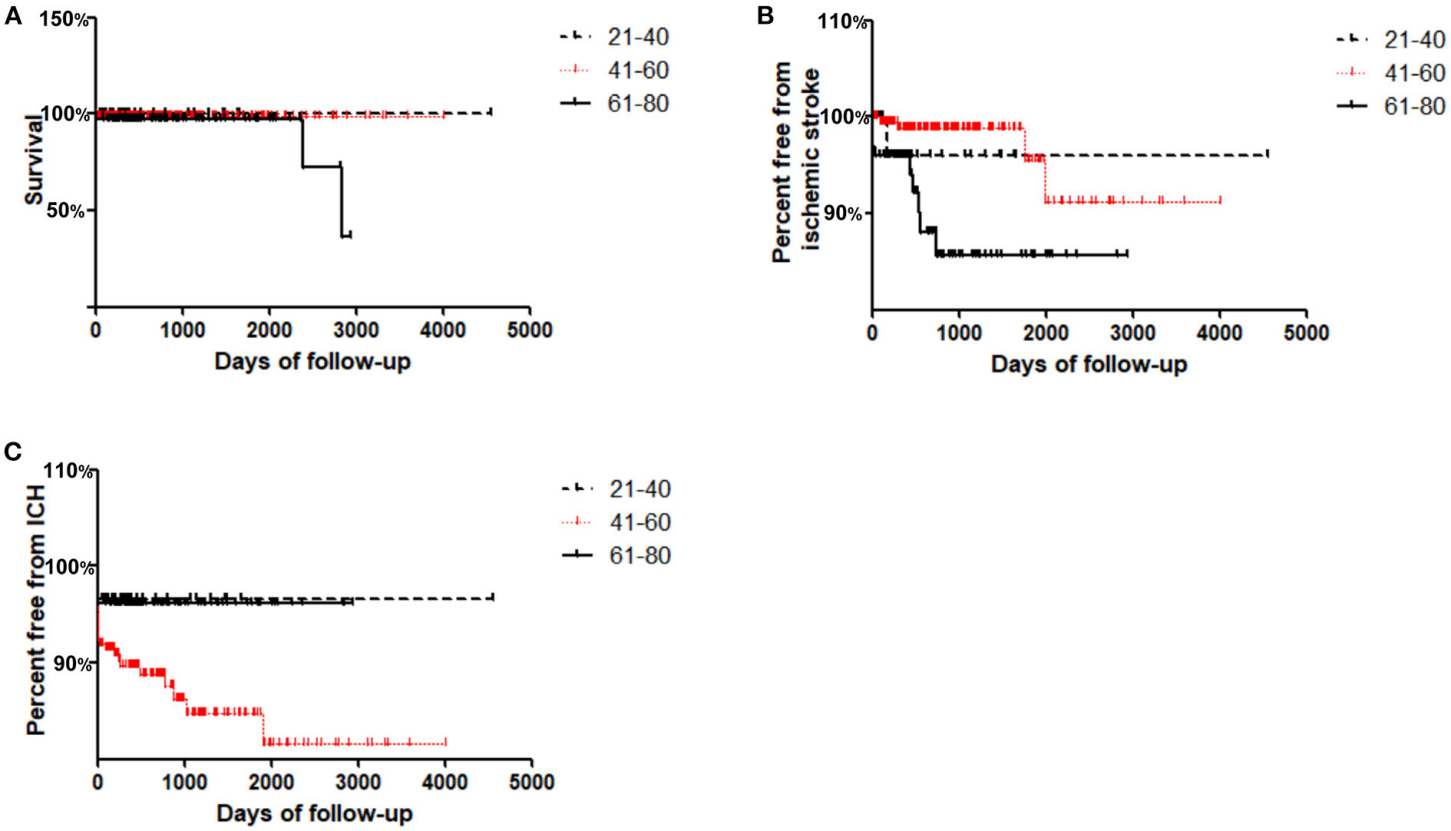
Kaplan–Meier curves for freedom from adverse events in Young (≤ 40 years), Middle (41–60 years) and Old (≥61 years) groups during follow-up. **(A)** Cumulative survival; **(B)** Freedom from ischemic stroke; **(C)** Freedom from ICH.

Long-term Kaplan–Meier curves are shown in Figure 1. The average/maximum length of follow-up for the young, middle and old groups was 1.8/12.5, 2.2/11.0, and 2.3/8.0 years, respectively. Composite survival was significantly lower at long-term follow-up for the old group (36.4%) than for the middle and young groups (99 and 100%, respectively; *P* = 0.027, Figure 1A). Freedom from ischemic stroke is shown in Figure 1B; only 85.6% of the old group had stroke-free survival by 8 years, which was lower than that of the middle and young groups (91.3 and 96.0%, respectively; *P* = 0.013, Figure 1B).

Interestingly, it was found that freedom from ICH in the middle group was lowest at the long-term follow-up (81.6%) compared with that in the young and old groups (96.7 and 96.1%, respectively; *P* = 0.04, Figure 1C).

## 4. Discussion

In this study, our data indicated that MCA stenting in elderly patients, especially for patients ≥61 years old, is associated with a high risk of adverse events, both at 90 days and long-term follow-up, and should be cautiously considered.

Endovascular treatment of symptomatic ICAS fell into disfavor after the prematurely terminated SAMMPRIS and VISSIT trials due to significantly higher periprocedural complications (2, 20). However, researchers in SAMMPRIS study also emphasized that further randomized trials assessing new techniques, or identifying any subgroup that may benefit from PTAS, was still needed. Therefore, neural interventional groups around the world are still trying to find a way to lower the procedural risk. Unfortunately, most of the studies focused on improving the clinical outcomes for patients with MCAS who received endovascular treatment by modifying endovascular techniques or selecting different stents (4, 21–23). Many other factors should have been put forward to explain the high complication rates, and further identification of risk factors can guide patient selection, which may also play an important role in reducing complications. Above all, in this study, inclusion and exclusion criteria were not changed by the results of SAMMPRIS. And aim of this study was identifying risk factors for MCA stenting, which might further help to identify any subgroup that may benefit from PTAS.

To date, vascular anatomy (24), age (12, 13, 24), neurointerventionist's experience (25) and sex (26) have been investigated to identify potential risk factors for endovascular treatment. Among these factors, age is one of the key factors that is widely studied in carotid artery stenting; however, the results are controversial. Chastain et al. reported a major adverse event rate of 25% in octogenarians vs. 8.2% in younger patients (27). In the CREST study, in 2004, Hobson et al. reported a 12% 30-day stroke rate and a 12% stroke and death rate in octogenarians who underwent carotid stenting. Nonoctogenarians had a 2.8% stroke rate and a 3.2% stroke or death rate (28). Stanziale et al. also reported that octogenarians undergoing carotid artery stenting are at higher risk than nonoctogenarians for periprocedural complications. Major event-free survival at 1 year is also significantly better in nonoctogenarians. They proposed that these risks should be weighed when considering carotid stenting in elderly patients (12). Unfortunately, Fanous et al. reported that vascular anatomy and not age is responsible for the increased risk of complications in symptomatic elderly patients undergoing carotid artery stenting (24). Although agreement is far from universal, in patients who underwent carotid stenting, the association of advanced age with worse outcome seems to be a persistent trend.

To the best of our knowledge, the effect of increased age on the safety and efficacy of the intervention for MCAS has not been reported previously. In the present study, we first found that the old and middle groups had more major adverse events ≤ 90 days after the stenting procedure than the young group, which indicated that advanced age is associated with increased 90-day complications. Further analysis revealed that advanced age increased the incidence of stent-related ischemic stroke and total 90-day complications (ischemic stroke, ICH and death).

By using univariable and multivariable analyses, we also confirmed that increased age was associated with increased 90-day ischemic stroke, and hypertension was also identified as a potential clinical risk factor for increased 90-day ischemic stroke in patients undergoing MCA stenting. Meanwhile, it was also demonstrated that aging was associated with 90-day mortality. These findings suggested that elderly patients with MCAS may be at a higher risk of 90-day complications with MCA stenting. For 90-day ICH, hypertension was found to be a potential clinical risk factor.

We also determined long-term outcomes in patients who underwent MCA stenting. Long-term survival curves in the old group in this study demonstrated a 36.4% survival rate at 8 years, which was significantly lower than that in the middle and young groups. Long-term freedom from ischemic stroke was also decreased in elderly patients who received MCA stenting. Interestingly, our data indicated that patients who received MCA stenting in the middle group (41–60 years old) had a higher incidence of ICH during the follow-up, which might be explained by the increased incidence of hypertension (compared with the young group) without compensatory mechanisms (compared with the old group) (29).

This study has a few limitations. First, this was a retrospective, single-center, nonrandomized study and suffers from selection bias. Second, the sample size for the young group was relatively small. A multicenter, prospective, controlled trial is still needed in the future to confirm our results and to determine the proper population that may benefit from MCA stenting.

## 5. Conclusion

Elderly patients undergoing MCA stenting are at higher risk than younger patients for 90-day complications, including ischemic stroke and death. Meanwhile, advanced age was also associated with decreased long-term survival and ischemic stroke-free survival during follow-up. Therefore, MCA stenting in elderly patients should be cautiously considered, especially for patients ≥61 years old.

## Data availability statement

The raw data supporting the conclusions of this article will be made available by the authors, without undue reservation.

## Ethics statement

The studies involving human participants were reviewed and approved by Medical Ethics Committee of the Second Affiliated Hospital, Army Medical University. The patients/participants provided their written informed consent to participate in this study.

## Author contributions

RX, GW, and JL drafted the manuscript and performed the statistical analyses in a blind way. SH, XZ, LY, FG, and YG collected the data. RX supervised throughout the study. All authors contributed to the article and approved the submitted version.
