# Supplementary material for: Advanced age is associated with increased adverse outcomes in patients undergoing middle cerebral artery stenting
Source: Front Neurol. 2023 Jan 18;13:1037034. doi: 10.3389/fneur.2022.1037034 (PMC9889558; doi:10.3389/fneur.2022.1037034)
Supplement: Supplementary file 1 [file Data_Sheet_1.docx]

***Supplementary Material***

**Advanced age is associated with increased adverse outcomes in patients undergoing middle cerebral artery stenting**

**Table S1. Medications of** **middle cerebral artery stent patients**

|  | **No. (%) of patients** | | | ***P**** | | |
| --- | --- | --- | --- | --- | --- | --- |
|  | **≤ 40 yrs**  **(n =30)** | **41-60 yrs**  **(n =210 )** | **≥ 61 yrs**  **(n = 108)** |  |  |  |
| **Preprocedure** |  |  |  | | |  |
| Aspirin | 2 (6.67 %) | 17 (8.10 %) | 14 (12.96 %) | | | 0.351 |
| Clopidogrel | 2 (6.67 %) | 11 (5.24 %) | 13 (12.04 %) | | | 0.071 |
| Nitrates | 0 (0.00) | 2 (0.95 %) | 0 (0.00) | | | 0.624 |
| Calcium-channel blockers | 3 (10.00 %) | 9 (4.29 %) | 23 (21.30 %) | | | 0.000 |
| β-blockers  **Follow-up**  **(within 3 months)** | 0 (0.00) | 0 (0.00) | 1 (0.93 %) | | | 0.397 |
| Aspirin + Clopidogrel | 27 (90.00 %) | 196 (93.33 %) | 96 (88.89 %) | | | 0.333 |
| Aspirin | 1 (3.33 %) | 3 (1.43 %) | 4 (3.70 %) | | | 0.293 |
| Clopidogrel | 3 (10.00 %) | 8 (3.81 %) | 5 (4.63 %) | | | 0.285 |
| Statin | 28 (93.33 %) | 204 (97.14 %) | 98 (90.74 %) | | | 0.042 |
| Nitrates | 0 (0.00 %) | 1 (0.48 %) | 3 (2.78 %) | | | 0.19 |
| Calcium-channel blockers | 2 (6.67 %) | 69 (32.86 %) | 37 (34.26 %) | | | 0.01 |
| β-blockers  **Late follow-up**  **(after 3 months)** | 0 (0.00) | 1 (0.48 %) | 5 (4.63 %) | | | 0.031 |
| Aspirin + Clopidogrel | 1 (3.33 %) | 5 (2.38 %) | 6 (5.56 %) | | 0.253 | |
| Aspirin | 14 (46.67 %) | 89 (42.38 %) | 39 (36.11 %) | | 0.444 | |
| Clopidogrel | 7 (23.33 %) | 83 (39.52 %) | 45 (41.67 %) | | 0.179 | |
| Statin | 20 (66.67 %) | 185 (88.10 %) | 86 (79.63 %) | | 0.006 | |
| Nitrates | 0 (0.00) | 0 (0.00) | 1 (0.93 %) | | 0.397 | |
| Calcium-channel blockers | 1 (3.33 %) | 31 (14.76 %) | 20 (18.52 %) | | 0.102 | |
| β-blockers | 1 (3.33 %) | 0 (0.00) | 3 (2.78 %) | | 0.026 | |

**Table S2. Major adverse events ≤** **90 days of stent procedure in patients with MCA occlusion (subgroup analysis)**

|  | **No. (%) of patients** | | | ***P**** | |
| --- | --- | --- | --- | --- | --- |
|  | **≤ 40 yrs**  **(n =3)** | **41-60 yrs**  **(n =25)** | **≥ 61 yrs**  **(n = 5)** |  |  |
| Ischemic stroke (stent related) | 0 (0.00) | 0 (0.00) | 0 (0.00) | | N/A |
| ICH | 0 (0.00) | 2(8.00 %) | 0 (0.00) | | N/A |
| TIA | 0 (0.00) | 0 (0.00) | 0 (0.00) | | N/A |
| [In-stent](javascript:;) [restenosis](javascript:;) (>50%) | 0 (0.00) | 0 (0.00) | 0 (0.00) | | N/A |
| MI | 0 (0.00) | 0 (0.00) | 0 (0.00) | | N/A |
| Death | 0 (0.00) | 1(4.00 %) | 0(0.00) | | N/A |
| Total | 0(3.33%) | 3(12.00%) | 0 (0.00) | | N/A |

^ICH, intra^[^cerebral^](javascript:;) [^hemorrhage^](javascript:;)^; TIA, transient ischemic attack; MI, myocardial infarction.^

**Table S3. Major adverse events ≤ 90 days of stent procedure in patients with MCA severe atherosclerotic stenosis (subgroup analysis)**

|  | **No. (%) of patients** | | | ***P**** | |
| --- | --- | --- | --- | --- | --- |
|  | **≤ 40 yrs**  **(n = 27)** | **41-60 yrs**  **(n = 185)** | **≥ 61 yrs**  **(n = 104)** |  |  |
| Ischemic stroke (stent related) | 0 (0.00) | 6 (3.24%) | 10 (9.62%) | | 0.043 |
| ICH | 1 (3.70%) | 27(14.59 %) | 8 (7.69) | | 0.102 |
| TIA | 0 (0.00) | 0 (0.00) | 0 (0.00) | | N/A |
| [In-stent](javascript:;) [restenosis](javascript:;) (>50%) | 0 (0.00) | 0 (0.00) | 0 (0.00) | | N/A |
| MI | 0 (0.00) | 0 (0.00) | 0 (0.00) | | N/A |
| Death | 0 (0.00) | 4(2.16%) | 8(7.69%) | | 0.049 |
| Total | 1(3.70%) | 37(20.00%) | 26 (25.00%) | | 0.037 |

^ICH, intra^[^cerebral^](javascript:;) [^hemorrhage^](javascript:;)^; TIA, transient ischemic attack; MI, myocardial infarction.^

**Table S4. Association among 90-day ischemic stroke and selected demographic, procedural, and clinical factors (subgroup analysis - MCA severe atherosclerotic stenosis)**

| **Factors** | **Univariable analysis** | |  | **Multivariable analysis^#^** | |
| --- | --- | --- | --- | --- | --- |
|  | **OR (95%CI)** | ***P*** |  | **OR (95%CI)** | ***P*** |
| **Age** | **1.069 (1.014-1.127)** | **0.012** |  | 1.061(0.997-1.130) | 0.063 |
| **Hypertension** | **8.730 (1.138-66.996)** | **0.037** |  | 6.211 (0.767-50.308) | 0.087 |
| CAD | 2.220 (0.872-5.652) | 0.094 |  | 2.031 (0.592-6.971) | 0.260 |
| Diabetes mellitus | 1.103 (0.390-3.119) | 0.854 |  | 1.032 (0.337-3.158) | 0.956 |
| Homocysteine | 0.954 (0.349-2.609) | 0.927 |  | Excluded |  |
| Hyperlipidemia | 1.782 (0.632-5.029) | 0.275 |  | 2.120 (0.675-6.660) | 0.198 |
| Smoking history | 0.770 (0.261-2.273) | 0.636 |  | 0.658 (0.145-2.998) | 0.589 |
| Drinking history | 0.826 (0.300-2.276) | 0.712 |  | 1.122 (0.262-4.811) | 0.877 |
| History of ischemic stroke | 2.967 (0.661-13.325) | 0.156 |  | 2.480 (0.324-18.995) | 0.382 |
| Symptomatic presentation | 1.250 (0.158-9.891) | 0.833 |  | 0.339 (0.022-5.275) | 0.440 |
| Aspirin use | 1.383 (0.299-6.397) | 0.678 |  | 3.290 (0.342-31.640) | 0.303 |
| Clopidogrel use | 0.800 (0.101-6.330) | 0.833 |  | 0.276 (0.015-4.988) | 0.383 |
| Time for qualifying event to stenting | 1.008 (0.996-1.020) | 0.217 |  | 1.007 (0.994-1.021) | 0.273 |

^CAD, Coronary artery disease; OR, Odds ratio; CI, Confidence interval.^

^# Adjusted for hypertension (or age), CAD, hyperlipidemia, smoking history, drinking history, history of ischemic stroke, symptomatic presentation, aspirin use, clopidogrel use, time for qualifying event to stenting.^

^Age was analyzed as an independent, continuous variable.^

**Table S5. Association among 90-day death and selected demographic, procedural, and clinical factors (subgroup analysis - MCA severe atherosclerotic stenosis)**

| **Factors** | **Univariable analysis** | |  | **Multivariable analysis^#^** | |
| --- | --- | --- | --- | --- | --- |
|  | **OR (95%CI)** | ***P*** |  | **OR (95%CI)** | ***P*** |
| **Age** | **1.081 (1.016-1.150)** | **0.013** |  | **1.126 (1.030-1.232)** | **0.009** |
| Hypertension | 1.092 (0.321-3.710) | 0.888 |  | 0.676 (0.159-2.870) | 0.595 |
| CAD | 1.717 (0.542-5.441) | 0.358 |  | 0.987 (0.212-4.594) | 0.986 |
| Diabetes mellitus | 1.876 (0.590-5.962) | 0.286 |  | 2.436 (0.650-9.128) | 0.187 |
| Homocysteine | 1.355 (0.421-4.362) | 0.611 |  | 0.982 (0.269-3.581) | 0.978 |
| Hyperlipidemia | 2.137 (0.630-7.248) | 0.223 |  | 3.574 (0.862-14.827) | 0.079 |
| Smoking history | 1.755 (0.552-5.572) | 0.340 |  | 1.017 (0.166-6.249) | 0.985 |
| Drinking history | 1.526 (0.474-4.913) | 0.479 |  | 1.861 (0.281-12.299) | 0.519 |
| History of TIA | 0.281 (0.036-2.216) | 0.228 |  | 0.713 (0.058-8.758) | 0.792 |
| History of ischemic stroke | 1.228 (0.325-4.643) | 0.762 |  | 0.852 (0.078-9.304) | 0.896 |
| Symptomatic presentation | 0.391 (0.081-1.899) | 0.244 |  | 0.160 (0.010-2.503) | 0.192 |
| Aspirin use | 3.407 (0.870-13.344) | 0.078 |  | 7.030 (0.658-75.102) | 0.107 |
| Clopidogrel use | 2.555 (0.527-12.389) | 0.244 |  | 0.471 (0.033-6.702) | 0.578 |
| Time for qualifying event to stenting | 0.999 (0.974-1.024) | 0.931 |  | Excluded |  |

^CAD, Coronary artery disease; TIA, transient ischemic attack; OR, Odds ratio; CI, Confidence interval.^

^# Adjusted for hypertension, CAD, diabetes mellitus, homocysteine, hyperlipidemia, smoking history, drinking history, history of TIA, history of ischemic stroke, symptomatic presentation, aspirin use, clopidogrel use.^

^Age was analyzed as an independent, continuous variable.^

**Table S6. Association among 90-day ICH and selected demographic, procedural, and clinical factors (subgroup analysis - MCA severe atherosclerotic stenosis)**

| **Factors** | **Univariable analysis** | |  | **Multivariable analysis^#^** | |
| --- | --- | --- | --- | --- | --- |
|  | **OR (95%CI)** | ***P*** |  | **OR (95%CI)** | ***P*** |
| **Hypertension** | **3.234 (1.209-8.652)** | **0.019** |  | **4.002 (1.422-11.265)** | **0.009** |
| Age | 0.986 (0.954-1.019) | 0.404 |  | 0.967 (0.928-1.009) | 0.121 |
| CAD | 0.818 (0.288-2.322) | 0.706 |  | 0.915 (0.318-2.631) | 0.870 |
| Diabetes mellitus | 0.807 (0.368-1.770) | 0.592 |  | 0.878 (0.377-2.046) | 0.763 |
| Homocysteine | 1.094 (0.526-2.276) | 0.810 |  | 1.168 (0.524-2.602) | 0.704 |
| Hyperlipidemia | 2.139 (0.994-4.601) | 0.052 |  | 2.099 (0.942-4.677) | 0.070 |
| Smoking history | 1.380 (0.659-2.891) | 0.393 |  | 2.142 (0.797-5.759) | 0.131 |
| Drinking history | 0.940 (0.452-1.956) | 0.869 |  | 0.646 (0.244-1.711) | 0.379 |
| History of TIA | 0.563 (0.209-1.519) | 0.257 |  | 0.588 (0.149-2.314) | 0.447 |
| History of ischemic stroke | 0.882 (0.400-1.945) | 0.756 |  | 0.729 (0.195-2.727) | 0.639 |
| Symptomatic presentation | 0.389 (0.134-1.124) | 0.081 |  | 0.471 (0.099-2.233) | 0.343 |
| Aspirin use | 0.981 (0.280-3.434) | 0.976 |  | Excluded |  |
| Clopidogrel use | 0.791 (0.177-3.531) | 0.759 |  | 0.953 (0.202-4.489) | 0.952 |
| Time for qualifying event to stenting | 0.996 (0.979-1.013) | 0.645 |  | 0.999 (0.983-1.015) | 0.886 |

^CAD, Coronary artery disease; TIA, transient ischemic attack; OR, Odds ratio; CI, Confidence interval.^

^# Adjusted for age, CAD, diabetes mellitus, homocysteine, hyperlipidemia, smoking history, drinking history, history of TIA, history of ischemic stroke, symptomatic presentation, aspirin use, clopidogrel use, time for qualifying event to stenting.^

^Age was analyzed as an independent, continuous variable.^
